# Anesthetic Stability of Propofol, Dexmedetomidine, and Isoflurane by Measuring Bispectral Index (BIS) and Hemodynamic Indices: A Comparative Study

**DOI:** 10.7759/cureus.24930

**Published:** 2022-05-11

**Authors:** Manoj Kumar, Atit Kumar, Jay Brijesh Singh Yadav, Sunil K Bhardwaj, Amit K Singh

**Affiliations:** 1 Anesthesiology, Uttar Pradesh University of Medical Sciences, Etawah, IND

**Keywords:** bispectral index, anesthetic stability, isoflurane, dexmedetomidine, propofol, hemodynamic indices

## Abstract

Background

Hemodynamic changes and anesthetic awareness occurring during surgery under general anesthesia is a great concern for both surgeon and anesthesiologist. Maintenance of the adequate depth of anesthesia throughout the intraoperative period is important in maintaining hemodynamic stability, preventing intraoperative awareness, and avoiding postoperative recall.

Aim

This study aims to predict the anesthetic stability of propofol, dexmedetomidine, and isoflurane by measuring bispectral index (BIS) and hemodynamic indices.

Materials and methods

This is a prospective comparative study. Sixty patients of either sex, aged 18-60 years, with American Society of Anesthesiologist (ASA) physical status classification I and II, undergoing elective surgical procedures requiring general anesthesia were allocated into three groups of 20 each. Patients in each group were administered standard general anesthesia with routine hemodynamic monitoring along with BIS, and values were recorded at baseline and thereafter at every five-minute interval for the duration of surgery. Anesthesia was maintained in Group P using a bolus dose of propofol 1 milligram.kg^-1^ for 10 minutes followed by propofol infusion 50-75 microgram.kg^-1^.minute^-1^, Group D with a bolus dose of dexmedetomidine 1 microgram.kg^-1^ for 10 minutes followed by infusion 0.2-0.7 microgram.kg^-1^.hour^-1^, and Group I with isoflurane at 1 minimum alveolar concentration (MAC) for 10 minutes and then maintained between 0.5 MAC and 1.5 MAC until the duration of surgery. To maintain the surgical plane of anesthesia, the BIS score was monitored between 40 and 65. The quantitative variables were expressed as mean±SD and compared between groups using Student’s unpaired t-test. Data analysis was done using SPSS Statistics for Windows version 20.0 (IBM Corp., Armonk, NY, USA). A p-value of <0.05 was considered statistically significant.

Results

During intergroup comparison among study drugs, the mean BIS values were statistically significant among the groups (p<0.05). Hemodynamic indices were significantly better maintained in the dexmedetomidine group as compared to the isoflurane and propofol groups throughout the intraoperative period (p<0.05).

Conclusion

Dexmedetomidine is better than propofol and isoflurane in maintaining the BIS score and hemodynamic parameters during the intraoperative period.

## Introduction

Anesthetic stability can be assessed by conventional methods such as heart rate (HR), blood pressure, pulse oximetry, respiratory rate, and clinical signs, e.g., perspiration, shedding of tears, and limb movement. Nowadays, to evaluate surgical plane anesthesia, electroencephalogram (EEG) and bispectral index (BIS) are used. The BIS is a continuous noninvasive electroencephalographic method that is used to monitor the hypnotic state during sedation and anesthesia [1-4]. The BIS is an effective tool that correlates well with brain metabolism and reflects accurately the response of the brain to a variety of anesthetic agents. It also enables anesthesiologists to titrate drugs and avoid adverse effects such as awareness and hemodynamic disturbances. Earlier studies [5-9] had been conducted between propofol and dexmedetomidine using the BIS and hemodynamic indices with the aim of evaluating dexmedetomidine as a sole anesthetic agent for maintaining the depth of anesthesia. Also, in the past, various inhalational agents [10-12] such as isoflurane, halothane, and sevoflurane had been studied for either consumption of inhalational agents or maintenance of the depth of anesthesia using the BIS. In the past, numerous studies have been done to assess the effects of various anesthetic drugs on intraoperative hemodynamic indices and the depth of anesthesia. We conducted this study to compare propofol, dexmedetomidine, and isoflurane, assessed their effect using the BIS and hemodynamic indices, and aimed to predict the anesthetic stability of the study drugs.

## Materials and methods

This prospective comparative study was conducted after obtaining approval from the institutional ethical committee (1664/UPUMS/Dean(M)/Ethical/2020-21/E.C. 79/2019-20) and informed consent from the patients. Sixty patients of either sex, aged 18-60 years, with American Society of Anesthesiologists (ASA) physical status classification I and II, undergoing elective surgical procedures requiring general anesthesia were enrolled for the study. Patients who refuse to participate, those with a known allergic reaction to any of the study medications, those with a history of psychiatric or neurological illness, pregnant and lactating women, those with a known history of convulsions or brain surgery, those with a history of alcohol and drug abuse, those with recent use of sedatives or analgesics, duration of surgery lasting more than 120 minutes, and those with hepatic, cardiac, pulmonary, and renal dysfunction were excluded. Sample size estimation was done by taking an alpha error of 0.05 and power of the study of 80% and considering a difference of 20% response rate among the groups to be significant. The total sample size came out to be 60 patients equally divided into three groups of 20 each. Patients were randomly allocated using a computer-generated number table into three groups: Group P (n=20), who received bolus 1 milligram.kg^-1^ propofol for 10 minutes followed by infusion (50-75 microgram.kg^-1^.minute^-1^); Group D (n=20), who received bolus dose of dexmedetomidine 1 microgram.kg^-1^ for 10 minutes followed by infusion (0.2-0.7 microgram.kg^-1^.hour^-1^); and Group I (n=20), who received 1 minimum alveolar concentration (MAC) of isoflurane for 10 minutes and then maintained between 0.5 MAC and 1.5 MAC.

After the informed written consent was taken, patients were kept fasting for six hours. Upon arrival in the operating room, ASA standard monitoring devices were implemented, and baseline values of heart rate (HR), mean arterial pressure (MAP), oxygen saturation (SpO2), end-tidal carbon dioxide (EtCO2), and BIS were recorded. Intravenous (IV) access was secured with an 18‑gauge cannula, and lactated Ringers solution was started at 6 ml.kg^-1^. The depth of anesthesia was recorded using a BIS monitor (BIS LoC 2 Channel, Covidien, Singapore). The sensors of the monitor were placed diagonally on the forehead after wiping the skin with alcohol and drying. One was placed at the center of the forehead approximately 2 inches above the bridge of the nose. The second and fourth were placed directly above the eyebrows and the third one on the temple between the corner of the eye and hairline. A standard anesthetic technique was used for all patients. Premedication was done with injection glycopyrrolate (5 microgram.kg^-1^), injection midazolam (50 microgram.kg^-1^), and injection fentanyl (2 microgram.kg^-1^) intravenously. After preoxygenation for three minutes, the patients were induced with injection thiopentone (5 milligram.kg^-1^) and relaxed with injection vecuronium (0.08-0.1 milligram.kg^-1^). Maintenance of anesthesia was done with 50% nitrous oxide in 50% oxygen along with study drugs according to study groups. Drugs were titrated according to respective group protocol to maintain a surgical plane of anesthesia with a BIS value between 40 and 65. Injection paracetamol (15 milligram.kg^-1^) was given for intraoperative analgesia in all the patients. Injection ondansetron (0.1 milligram.kg^-1^) was given half an hour before the expected completion of surgery. Anesthetic stability was evaluated using intraoperative hemodynamic parameters such as HR, MAP, SpO2, EtCO2, and BIS value recorded at the start of surgery and thereafter at five-minute intervals until the end of surgery. Residual neuromuscular blockade was reversed with injection neostigmine (0.05 milligram.kg^-1^) and injection glycopyrrolate (0.01 milligram.kg^-1^). The duration of surgery was considered from skin incision to last skin suture. Any episode of hypertension, tachycardia, patient movement, grimacing, lacrimation, or sweating during the maintenance period was defined as inadequate anesthesia. Any sign of inadequate anesthesia despite the target BIS value was treated by an escalation of drug concentration as defined in the groups.

Statistical analysis

Quantitative variables were expressed as mean±SD. Quantitative variables were compared between groups using Student’s unpaired t-test. A p-value of <0.05 was considered statistically significant. Data entry and analysis were done using MS Excel (Microsoft Corp., Redmond, WA, USA) and SPSS Statistics for Windows version 20.0 (IBM Corp., Armonk, NY, USA).

## Results

**Figure 1:**
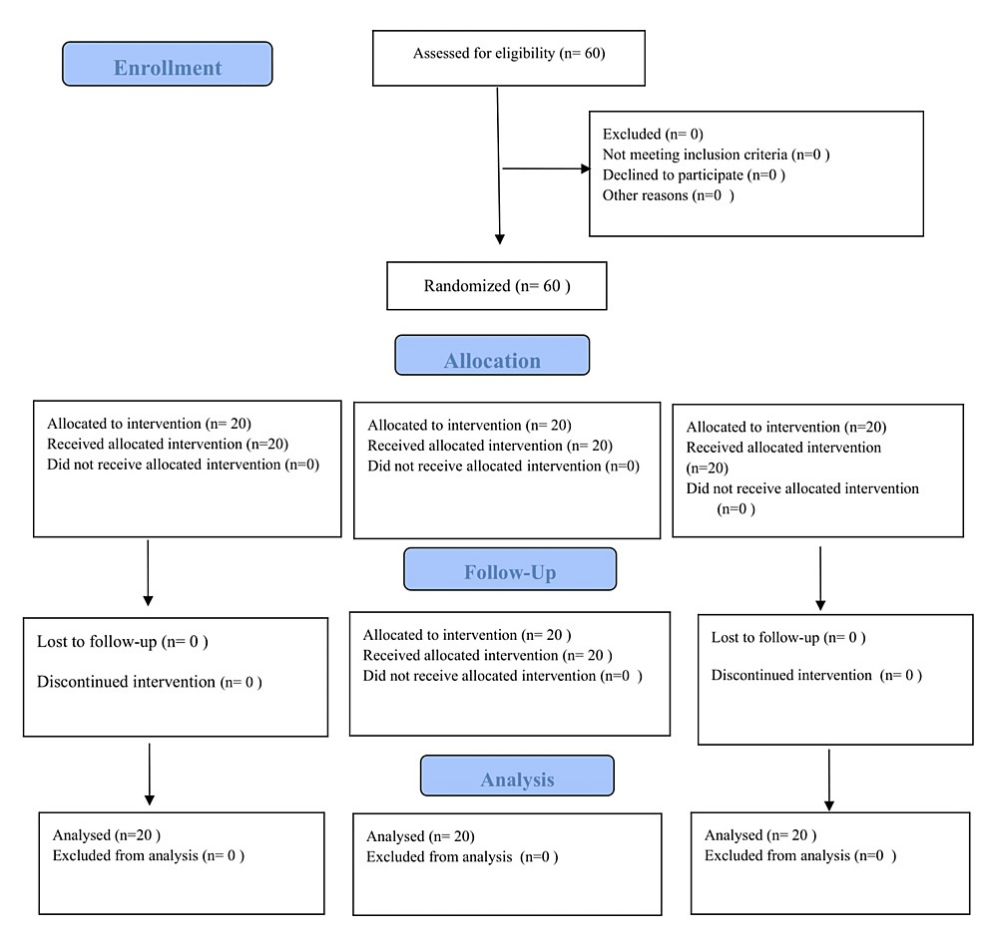
Consort flow diagram

None of the patients were excluded from our study (Figure 1). A total of 60 patients were enrolled in the study divided into three groups of 20 each.

The demographic characteristics (age, gender, and ASA physical status classification) were comparable between the groups (p>0.05) (Table 1). The mean duration of surgery was 74.50±19.73, 72.25±10.82, and 77.25±14.09 minutes in the isoflurane, propofol, and dexmedetomidine groups, respectively (Table 1). On intergroup comparisons, no statistically significant difference was observed between the groups (p>0.05).

**Table 1:** Demographic data of the studied groups

Age (years)	Isoflurane (n=20) (mean±SD)	Propofol (n=20) (mean±SD)	Dexmedetomidine (n=20) (mean±SD)	p-value		
				I versus P	I versus D	P versus D
	32.25±10.51	37.40±13.16	33.65±9.48	0.090	0.330	0.154
Gender	Number (%)	Number (%)	Number (%)	p-value		
Male	4 (20)	8 (40)	8 (40)	0.301		
Female	16 (80)	12 (60)	12 (60)			
American Society of Anesthesiologists Physical Status (ASA-PS)	Number of patients	Number of patients	Number of patients	p-value		
ASA-PS grade I/II	18/2	14/6	16/4	0.287		
Duration of surgery in the studied groups (minutes)	Isoflurane (n=20) (mean±SD)	Propofol (n=20) (mean±SD)	Dexmedetomidine (n=20) (mean±SD)	p-value		
				I versus P	I versus D	P versus D
	74.50±19.73	72.25±10.82	77.25±14.09	0.329	0.307	0.108

Throughout the intraoperative period, the mean heart rate was less in the dexmedetomidine group, followed by the isoflurane, and it was higher in the propofol group (Table 2). During the intergroup comparison, the mean heart rate values were observed to be higher in the propofol group compared to the dexmedetomidine group and remained statistically significant at all time periods. When isoflurane was compared to dexmedetomidine, the mean heart rate values remained significantly less in the dexmedetomidine group (p<0.05). However, the heart rate values remained statistically not significant between the propofol group and the isoflurane group at all time periods (p>0.05).

**Table 2:** Comparison of mean heart rate (HR) among the groups across the time periods

Heart rate	Isoflurane (n=20)		Propofol (n=20)		Dexmedetomidine (n=20)		p-value		
	Mean	±SD	Mean	±SD	Mean	±SD	I versus P	I versus D	P versus D
Baseline	85.20	±3.53	85.05	±3.89	85.90	±4.55	0.450	0.295	0.265
5 minutes	85.10	±5.83	86.60	±7.43	80.15	±5.48	0.241	0.004	0.002
10 minutes	87.00	±6.39	89.45	±8.53	77.30	±5.58	0.155	<0.001	<0.001
15 minutes	88.75	±8.19	92.10	±6.96	79.05	±4.65	0.086	<0.001	<0.001
20 minutes	88.90	±9.94	91.45	±6.88	79.30	±5	0.176	<0.001	<0.001
25 minutes	88.75	±10.63	90.90	±5.77	77.05	±4.51	0.216	<0.001	<0.001
30 minutes	88.90	±9.79	89.85	±7.9	78.30	±5.28	0.369	<0.001	<0.001
35 minutes	87.95	±10.74	87.80	±8.32	77.40	±5.66	0.480	<0.001	<0.001
40 minutes	88.35	±10.58	89.35	±6.74	77.95	±4.48	0.362	<0.001	<0.001
45 minutes	88.80	±11.3	89.80	±8.58	78.75	±5.04	0.377	<0.001	<0.001
50 minutes	86.05	±8.77	88.25	±7.22	78.05	±5.11	0.196	<0.001	<0.001
55 minutes	86.85	±8.07	89.65	±7.56	80.35	±4.34	0.132	0.001	<0.001
60 minutes	85.94	±10.95	89.72	±7.96	80.56	±5.22	0.127	0.036	<0.001
65 minutes	87.21	±10.42	88.71	±7.55	81.53	±4.72	0.324	0.026	0.001
70 minutes	86.80	±9.81	89.46	±7.08	81.57	±4.09	0.229	0.043	<0.001
75 minutes	89.63	±14.04	87.82	±5.98	80.33	±4.33	0.353	0.022	0.001
80 minutes	88.83	±16.9	88.50	±8.39	81.22	±4.63	0.486	0.108	0.032
85 minutes	84.60	±18.28	90.00	±5.66	81.86	±4.34	0.356	0.353	0.030
90 minutes	87.40	±17.05	91.50	±4.95	83.00	±1.15	0.382	0.314	0.011
95 minutes	85.75	±18.84	86.00	-	80.00	±0	-	0.352	-
100 minutes	88.00	±10	82.00	-	78.00	-	-	-	-
105 minutes	99.50	±3.54	-	-	86.00	-	-	-	-
110 minutes	94.50	±2.12	-	-	80.00	-	-	-	-
115 minutes	99.00	±7.07	-	-	82.00	-	-	-	-
120 minutes	95.00	-	-	-	-	-	-	-	-

Throughout the intraoperative period, the mean arterial pressure (MAP) was less in the dexmedetomidine group, followed by isoflurane, and it was higher in the propofol group (Table 3). During the intergroup comparison, MAP values were found to be higher in the propofol group compared to the dexmedetomidine group and remained statistically not significant during the first 10 minutes and thereafter remained statistically significant at all time periods (p<0.05). When isoflurane was compared to dexmedetomidine, MAP values remained significantly less in the dexmedetomidine group (p<0.05). However, the MAP values remained statistically not significant between the propofol group and the isoflurane group at all time periods during the intraoperative period (p>0.05).

**Table 3:** Comparison of mean arterial pressure (MAP) among the groups across the time periods

Mean arterial pressure	Isoflurane		Propofol		Dexmedetomidine		p-value		
	Mean	±SD	Mean	±SD	Mean	±SD	I versus P	I versus D	P versus D
Baseline	96.85	±7.04	97.55	±4.94	97.75	±2.81	0.359	0.299	0.438
5 minutes	94.95	±5.69	96.05	±4.43	94.95	±4.9	0.250	0.500	0.231
10 minutes	93.30	±5.88	94.35	±4.68	94.00	±6.48	0.268	0.361	0.423
15 minutes	94.95	±5.15	94.95	±5.08	89.70	±8.58	0.500	0.012	0.012
20 minutes	96.90	±4.71	95.10	±4.17	86.00	±6.3	0.104	<0.001	<0.001
25 minutes	96.00	±5.47	95.05	±4.82	85.90	±6.61	0.282	<0.001	<0.001
30 minutes	95.50	±5.3	96.40	±4.67	86.15	±5.71	0.286	<0.001	<0.001
35 minutes	95.60	±5.6	96.30	±4.39	86.30	±5.5	0.331	<0.001	<0.001
40 minutes	93.45	±7.29	96.60	±4.19	88.57	±5.93	0.051	0.013	<0.001
45 minutes	95.25	±6.02	96.65	±3.3	87.55	±5.63	0.184	<0.001	<0.001
50 minutes	96.00	±6.93	97.55	±3.76	87.95	±6.75	0.192	<0.001	<0.001
55 minutes	97.85	±6.95	98.20	±3.87	89.73	±6.21	0.423	<0.001	<0.001
60 minutes	96.75	±5.05	97.44	±4.29	91.11	±5.39	0.334	0.002	<0.001
65 minutes	98.50	±6.39	98.76	±5.31	88.88	±6.66	0.450	<0.001	<0.001
70 minutes	99.60	±7.63	100.00	±4.22	89.43	±4.73	0.437	<0.001	<0.001
75 minutes	100.88	±4.94	101.82	±3.68	90.25	±6.44	0.319	<0.001	<0.001
80 minutes	101.17	±5.04	101.25	±5.19	88.56	±5.2	0.490	<0.001	<0.001
85 minutes	101.00	±6.28	98.00	±4.24	90.71	±5.94	0.286	0.008	0.078
90 minutes	101.80	±5.89	103.00	±1.41	88.75	±4.65	0.399	0.004	0.008
95 minutes	100.00	±5.77	102.00	-	94.00	±2.83	-	0.127	-
100 minutes	99.00	±7.55	107.00	-	90.00	-	-	-	-
105 minutes	99.00	±7.07	-	-	102.00	-	-	-	-
110 minutes	98.00	±5.66	-	-	97.00	-	-	-	-
115 minutes	101.50	±3.54	-	-	103.00	-	-	-	-
120 minutes	103.00	-	-	-	-	-	-	-	-

The mean SpO2 values remained similar at all time intervals in all the groups. During intergroup comparisons between the various groups, mean values were observed to be statistically not significant during the intraoperative periods (p>0.05) (Table 4).

**Table 4:** Intergroup comparison of changes in arterial oxygenation saturation (SpO2) across the time periods

SpO2	Isoflurane		Propofol		Dexmedetomidine		p-value		
	Mean	±SD	Mean	±SD	Mean	±SD	I versus P	I versus D	P versus D
Baseline	99.50	±0.69	99.35	±0.59	99.50	±0.61	0.231	0.500	0.216
5 minutes	99.45	±0.69	99.45	±0.69	99.65	±0.59	0.500	0.164	0.164
10 minutes	99.60	±0.5	99.50	±0.51	99.60	±0.5	0.269	0.500	0.269
15 minutes	99.45	±0.6	99.60	±0.6	99.45	±0.69	0.218	0.500	0.233
20 minutes	99.60	±0.5	99.30	±0.73	99.55	±0.51	0.070	0.378	0.109
25 minutes	99.50	±0.69	99.25	±0.64	99.45	±0.6	0.121	0.404	0.158
30 minutes	99.60	±0.5	99.55	±0.6	99.50	±0.61	0.389	0.287	0.398
35 minutes	99.60	±0.6	99.50	±0.69	99.50	±0.61	0.313	0.301	0.500
40 minutes	99.55	±0.69	99.55	±0.51	99.30	±0.8	0.500	0.148	0.123
45 minutes	99.50	±0.61	99.50	±0.61	99.35	±0.67	0.500	0.231	0.231
50 minutes	99.50	±0.51	99.55	±0.6	99.50	±0.61	0.390	0.500	0.398
55 minutes	99.60	±0.5	99.55	±0.6	99.60	±0.6	0.389	0.500	0.397
60 minutes	99.50	±0.63	99.56	±0.62	99.56	±0.51	0.399	0.389	0.500
65 minutes	99.29	±0.83	99.41	±0.62	99.47	±0.72	0.315	0.255	0.400
70 minutes	99.40	±0.52	99.46	±0.52	99.50	±0.52	0.390	0.323	0.424
75 minutes	99.25	±0.71	99.64	±0.5	99.67	±0.65	0.091	0.096	0.451
80 minutes	99.17	±0.75	99.25	±0.5	99.56	±0.53	0.426	0.129	0.175
85 minutes	99.60	±0.55	100.00	±0	99.86	±0.38	0.187	0.178	0.313
90 minutes	99.20	±0.45	99.50	±0.71	99.75	±0.5	0.257	0.062	0.316
95 minutes	99.75	±0.5	99.00	-	99.00	±1.41	-	0.178	-
100 minutes	99.67	±0.58	98.00	-	99.00	-	-	-	-
105 minutes	99.50	±0.71	-	-	100.00	-	-	-	-
110 minutes	99.50	±0.71	-	-	100.00	-	-	-	-
115 minutes	99.00	±1.41	-	-	99.00	-	-	-	-
120 minutes	99.00	-	-	-	-	-	-	-	-

**Figure 2:**
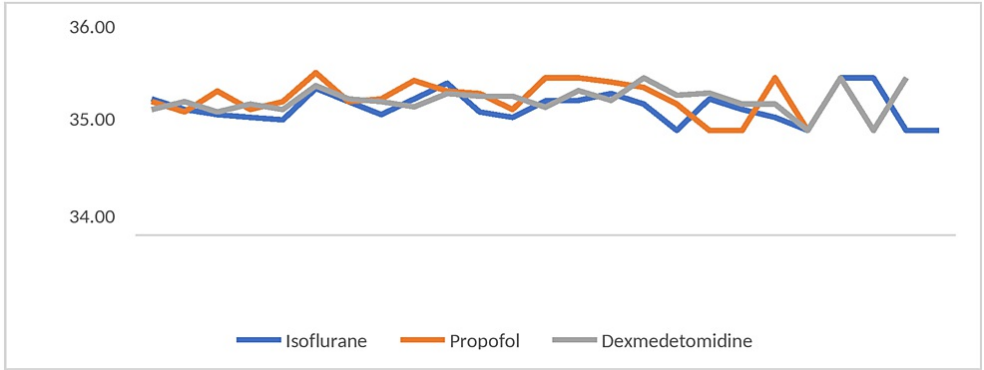
Intergroup comparison of changes in end-tidal capnography (EtCO2) across the time periods

The mean EtCO2 values remained comparable at all time intervals in all the groups. During intergroup comparisons, mean values were observed to be statistically not significant during the intraoperative periods (p>0.05) (Figure 2).

**Figure 3:**
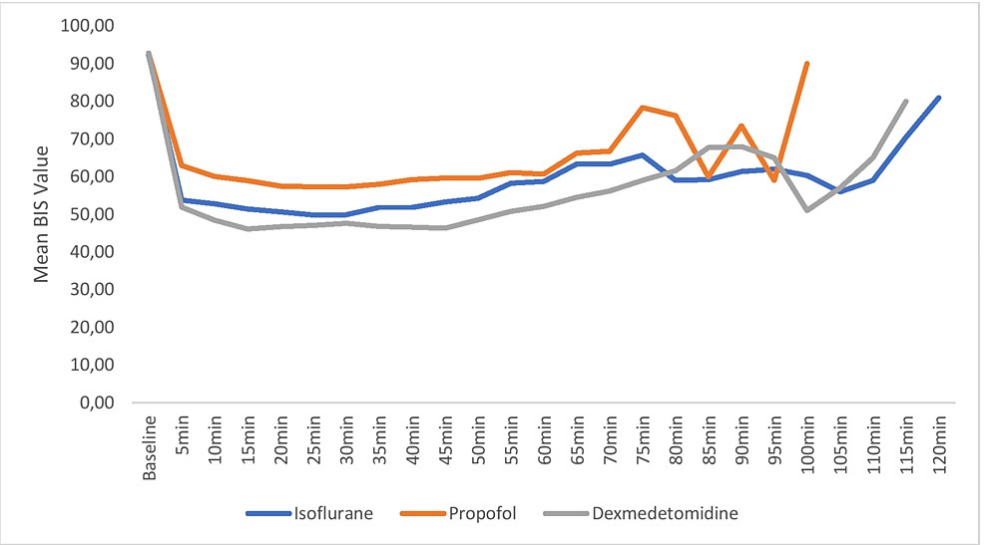
Comparison of bispectral index (BIS) scores among the groups across the time periods

The mean BIS scores in the isoflurane (I), propofol (P), and dexmedetomidine (D) groups at baseline were 92.45±0.94, 92.75±1.12, and 92.85±1.18, respectively, and were comparable between the groups (Figure 3). The mean BIS values were lower in the dexmedetomidine group compared to the propofol and isoflurane groups during the intraoperative period. During the intergroup comparison, the mean BIS values were significantly less in the dexmedetomidine group compared to the propofol group for the first 75 minutes and thereafter remained statistically not significant throughout the intraoperative period. When dexmedetomidine was compared with isoflurane, the mean BIS values were lower in the dexmedetomidine group and statistically significant for the initial 65 minutes. However, when isoflurane was compared with propofol, the mean BIS values were observed to be lower in the isoflurane group compared to the propofol group for the initial 50 minutes and then at 75th minute and 80th minute during the intraoperative period (p<0.05).

## Discussion

General anesthesia is a balance between anesthetic drug requirements and the state of arousal of the patient. During general anesthesia, inadvertent underdosing can cause an increased incidence of awareness, or overdosing can lead to hemodynamic instability, delayed recovery, and other complications [13]. In the current scenario, total intravenous anesthesia (TIVA) is gaining more and more importance in the anesthetic practice. The combination of dexmedetomidine and propofol forms an integral limb in TIVA. Our study demonstrates the promise of these drugs in maintaining stable intraoperative hemodynamic depth coupled with prompt postoperative awakening. In our study, hemodynamic parameters such as heart rate and mean arterial pressure were more stable in the dexmedetomidine group, followed by the isoflurane and propofol groups. This is consistent with the study done by Khare et al. [14] who observed more stable hemodynamic parameters with dexmedetomidine compared to propofol in a prospective, randomized, double‑blinded clinical study. Similarly, Tripathi et al. [15] conducted a prospective observational study on 28 patients allocated into two groups of 14 each and reported better hemodynamic parameters in patients in the dexmedetomidine group compared to the midazolam group. Similar observations were also reported by Ar et al. [16] and Patel et al. [17] in a prospective, randomized, placebo-controlled study. Tang et al. [18] studied the neuroprotective effect of BIS-guided fast-track anesthesia using sevoflurane combined with dexmedetomidine (DS) for intracranial aneurysm embolization. It was observed that the DS group had stable blood pressure as compared to the normal saline group during the intraoperative period and awakening from anesthesia. Similar observations were also reported in our study. Sen et al. [19] conducted a prospective, randomized, double-blinded, placebo-controlled, open-label study and reported that MAP was better maintained in the dexmedetomidine group.

In our study, the mean BIS values were observed to be lower in the dexmedetomidine group compared to the isoflurane and propofol groups across all time periods. However, BIS values were lower in the isoflurane group compared to the propofol group. During emergence from anesthesia, patients in the propofol group had a better postoperative awakening as compared to the isoflurane and dexmedetomidine groups. Dexmedetomidine had a better awakening than the isoflurane group during postoperative recovery. This is in line with the prospective, randomized study conducted by Chattopadhyay et al. [20] on 60 patients. They reported that dexmedetomidine infusion showed better maintenance of the depth of anesthesia when compared with propofol. Another study done by Abd El-Hamid et al. [21] during cesarean section under general anesthesia reported that the mean BIS value and hemodynamic indices in the dexmedetomidine group were lower than low-flow isoflurane group, which is similar to our study. The effects of bispectral index monitoring on isoflurane consumption and recovery profiles for anesthesia by Shafiq et al. [22] in 60 patients showed that the use of BIS resulted in a 40% reduction in isoflurane usage; also, patients having BIS monitoring awoke earlier and had better recovery profiles at the end of anesthesia. Venn et al. [23] evaluated the efficacy of dexmedetomidine in an intensive care unit on 12 patients and reported more sedation in the dexmedetomidine group but without any delay in extubation. Similar observations were seen in our study, where patients in the dexmedetomidine group were sedated without impairment in ventilation when compared to the propofol group, where they were completely awake. No complication due to any of our study drugs, recall, dreaming, and any sign of inadequate anesthesia was observed in our study patients during or after the surgery evaluated until 24 hours postoperatively. There are a few limitations to our study. This study could have been performed with larger sample size; the high cost of the BIS monitor and electrodes is also a limiting factor. We did not measure the plasma levels of the study drugs and MAC values of the inhalational agents.

## Conclusions

During general anesthesia, maintenance of the adequate depth and stable hemodynamic parameters are necessary to prevent awareness and reduce stress response and possible autonomic nervous system instability associated with surgery. In this clinical trial, we concluded that dexmedetomidine is more effective in maintaining the anesthetic stability during the intraoperative periods compared to isoflurane and propofol. So, for maintenance of the depth of anesthesia, dexmedetomidine can be used as the sole agent.
